# Supplementary material for: Highly and Lowly Domesticated Endangered Fish From a Conservation Hatchery Diverge in Their Thermal Physiology, Transcriptome, and Methylome
Source: Evol Appl. 2026 Mar 18;19(3):e70220. doi: 10.1111/eva.70220 (PMC13093269; doi:10.1111/eva.70220)
Supplement: Supplementary file 1 — Figure S1: Box plot distribution of each individual offspring's numerical domestication index (DI) number in each low and high DI category. Figure S2: Percent recovered offspring per family reared at either 15°C (blue) or 18°C (orange). Figure S3: Temperature by Domestication Index (DI) interaction effects on gene expression (red colors represents higher expression and blue colors represents lower expression). Figure S4: Venn diagram of DMRs due to acclimation temperature subsetted for low DI fish only and high DI fish only. Figure S5: Venn diagram of DMRs due to domestication index found in our study (blue) compared to DMRs found in Habibi et al. 2024 (yellow). Figure S6: Venn diagram of DMRs due to rearing temperature (blue) found in our study compared to DMRs found in Habibi et al. 2024 (yellow). Figure S7: Histogram of Weir pairwise F ST (window size of 10,000 bp and a sliding window of 5000 bp) between low and high DI progenitors. Table S1: HOMER annotation of hypermethylated DMRs due to acclimation temperature. Table S2: HOMER annotation of hypomethylated DMRs due to acclimation temperature. Table S3: HOMER annotation of hypermethylated DMRs due to domestication index (DI). Table S4: HOMER annotation of hypomethylated DMRs due to domestication index (DI). Table S5: Genes that showed a significant correlation (with Bonferroni correction applied) between expression levels and percent methylation for DEG and DMRs due to acclimation temperature. Table S6: Genes that showed a significant correlation (with Bonferroni correction applied) between expression levels and percent methylation for DEG and DMRs due to domestication index (DI). [file EVA-19-e70220-s001.docx]

**SUPPLEMENTAL FIGURES and TABLES**

**Epigenetic and transcriptomic responses to warming are similar between highly and lowly domesticated Delta smelt from a conservation hatchery**

Joanna S. Griffiths^1,2^, Amanda J. Finger^3^, Melinda R. Baerwald^4^, Md Mostafizur Rahman^5^, Tien-Chieh Hung^5^, Nann A. Fangue^6*^, and Andrew Whitehead^1*^


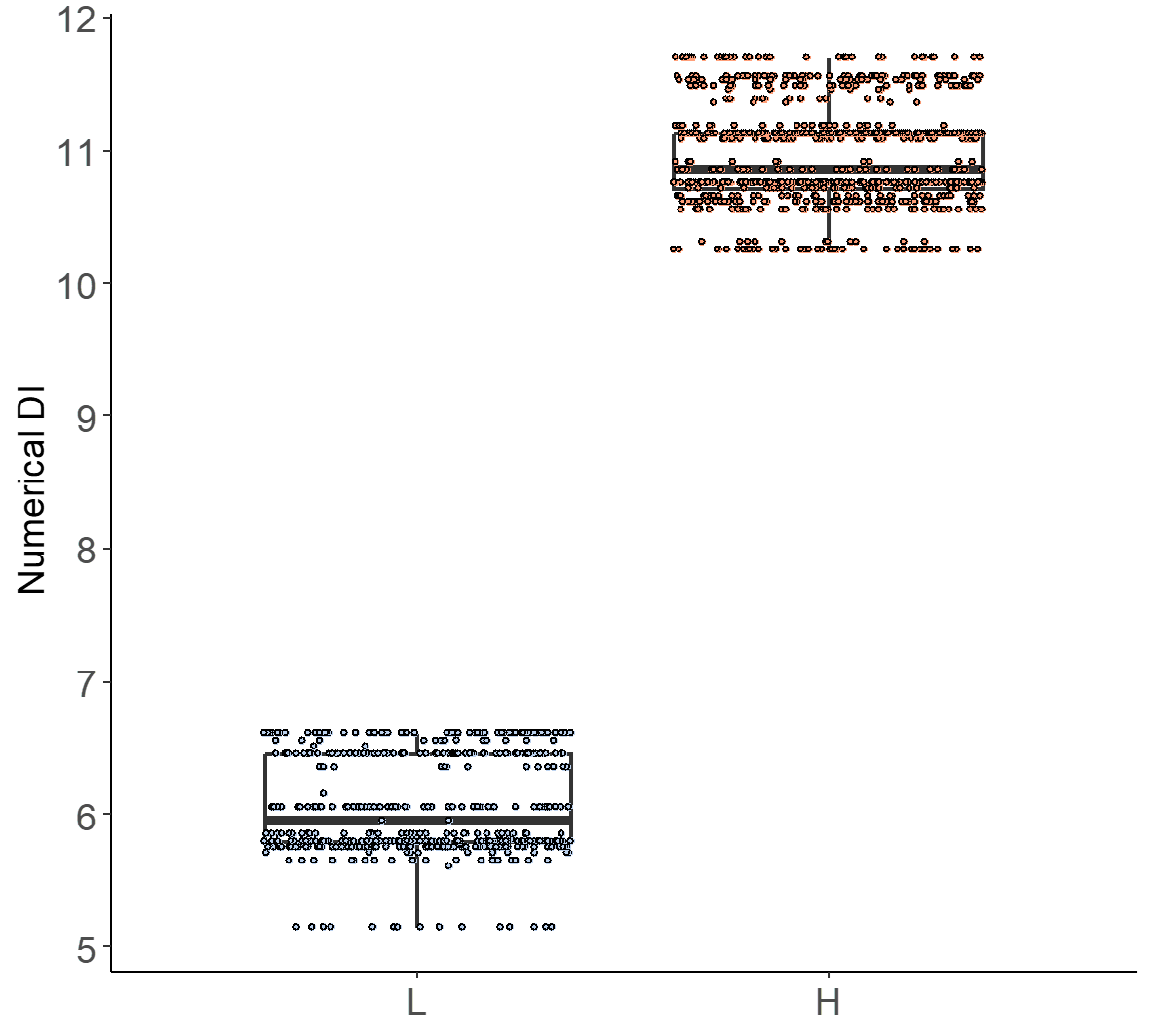


Figure S1: Box plot distribution of each individual offspring’s numerical domestication index (DI) number in each low and high DI category. In FCCL, low DI fish are defined as having a numerical DI below 7 and high DI fish are defined as having a numerical DI above 10. Box plot displays median (middle line of box), the first and third quartile of data (box edges), and full range of data (whiskers). Mean numerical DI in the low group was 6.06 and the high group was 10.96


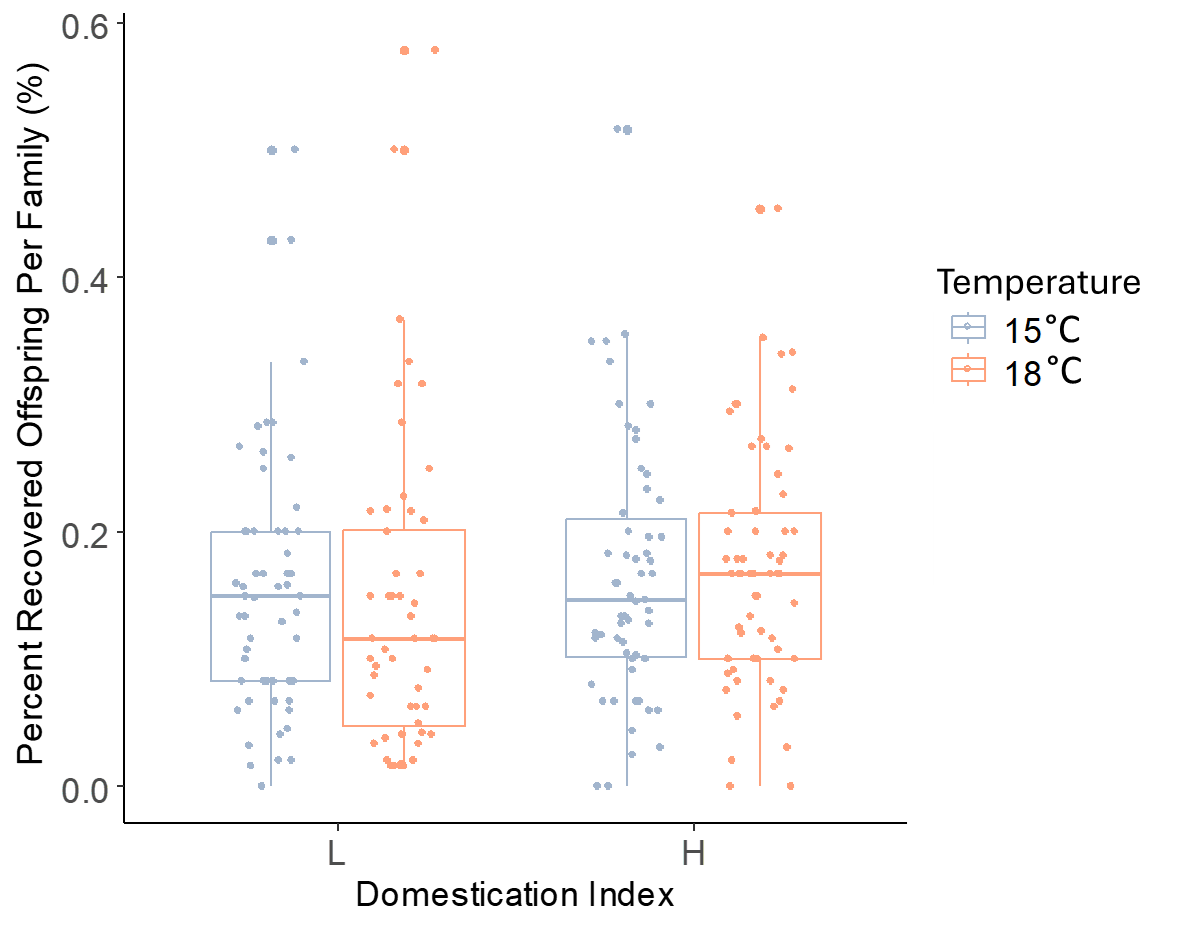


Figure S2: Percent recovered offspring per family reared at either 15°C (blue) or 18°C (orange). Each family is grouped by is domestication index (DI) as either low or high. There were no differences in family survival between rearing temperatures, nor DI. Box plot displays median (middle line of box), the first and third quartile of data (box edges), and full range of data (whiskers).


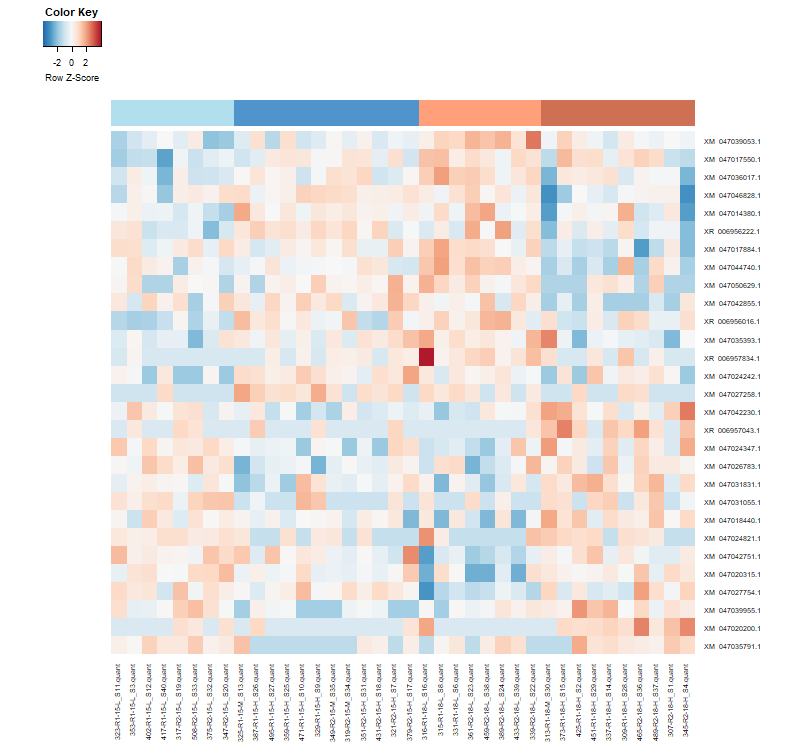


15℃

18℃

Low DI

High DI

Low DI

High DI

Figure S3: Temperature by Domestication Index (DI) interaction effects on gene expression (red colors represents higher expression and blue colors represents lower expression). Heatmap of expression patterns for genes that were significantly differentially expressed. Each column represents an individual from either a high or low DI background, reared at either 15°C  or 18°C . Rows are sorted by gene expression level and each row is z-score normalized.


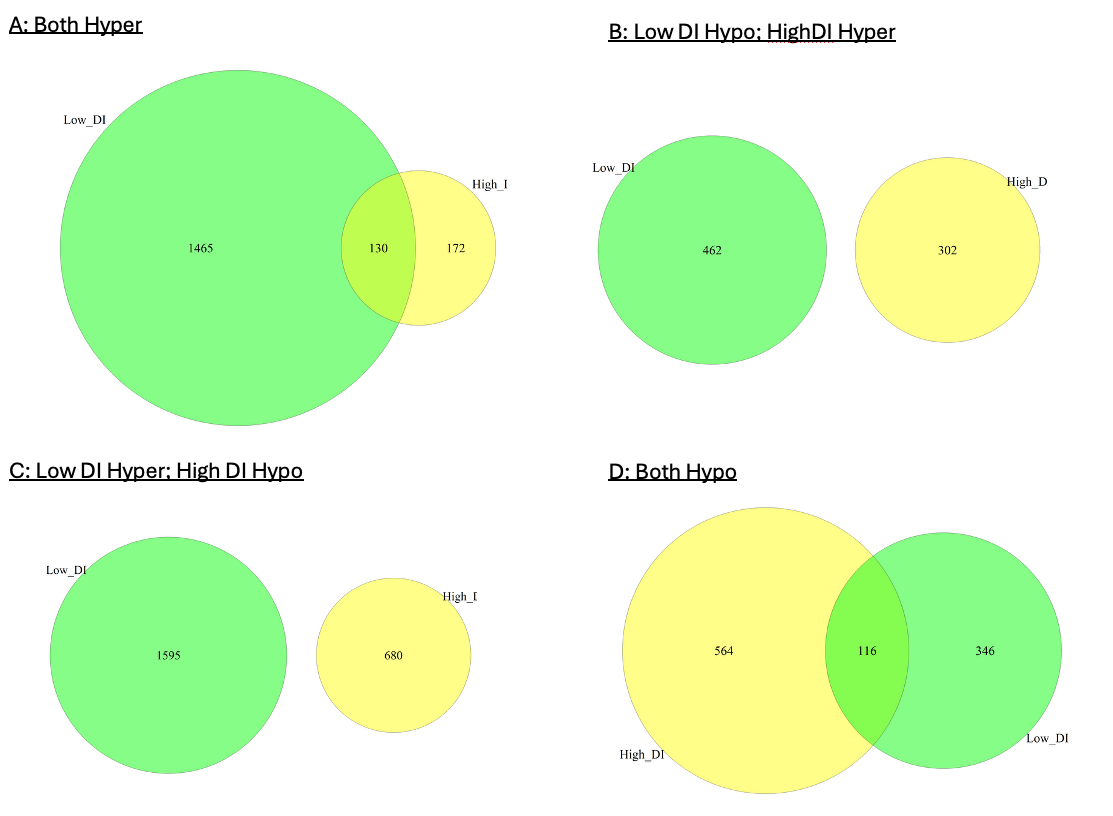


Figure S4: Venn diagram of DMRs due to acclimation temperature subsetted for low DI fish only and high DI fish only. We then identified DMRs that were hypermethylated in both datasets (A), hypomethylated in low DI fish and hypermethylated in high DI fish (B), hypermethylated in low DI fish, hypomethylated in high DI fish (C), and hypomethylated in both datasets (D). An interaction effect of acclimation temperature and DI was defined as any DMRs that were hypermethylated in one group and hypomethylated in the other group. Using this approach, we did not find any overlap of DMRs that were hypermethylated in one group and hypomethylated in the other group (B and C).


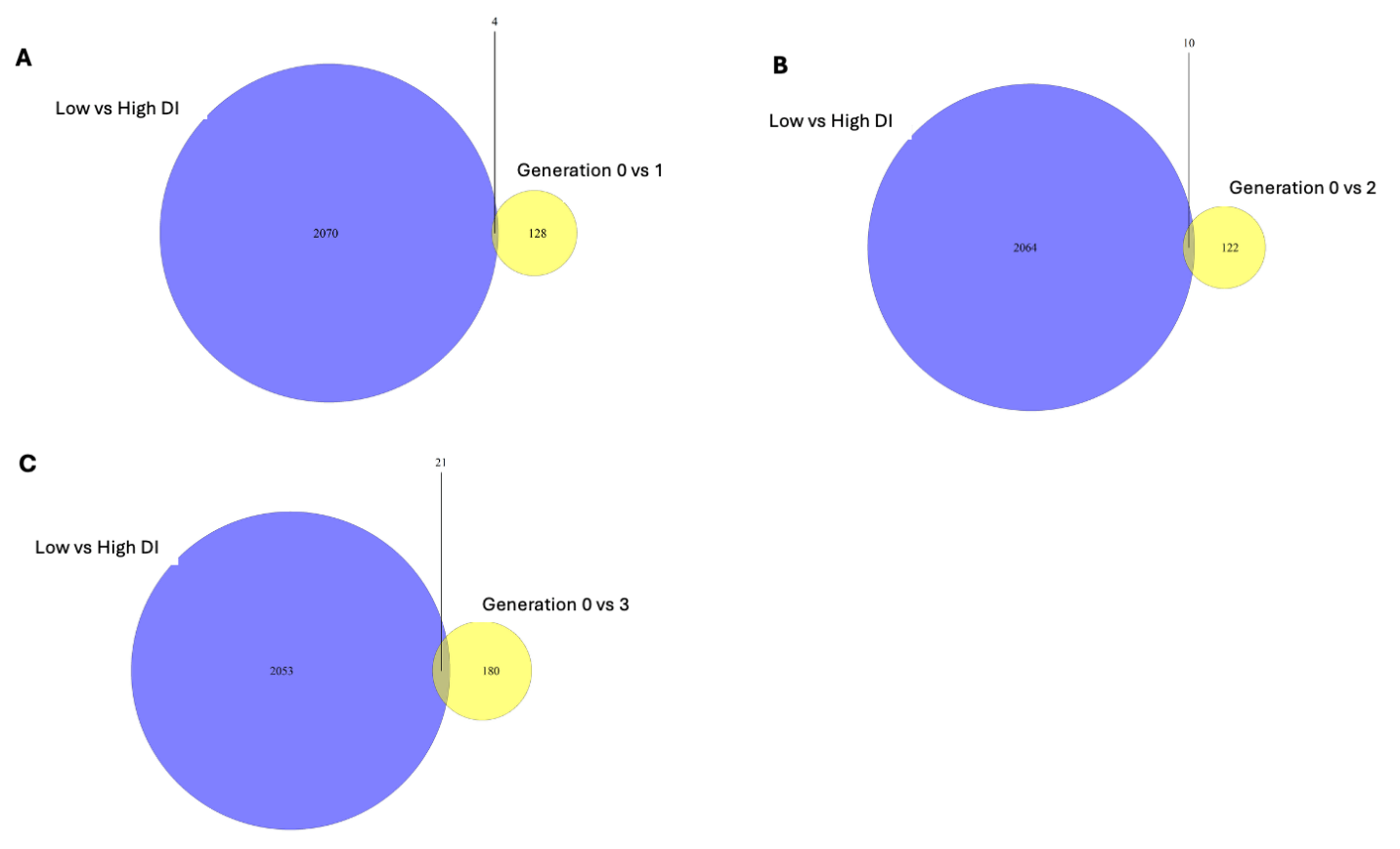


Figure S5: Venn diagram of DMRs due to domestication index found in our study (blue) compared to DMRs found in Habibi et al. 2024 (yellow).  Overlap of DMRs between low and high DI fish and those between generation 0 to generation 1 from Habibi et al. 2024 (A). Overlap of DMRs between low and high DI fish and those between generation 0 to generation 2 from Habibi et al. 2024 (B). Overlap of DMRs between low and high DI fish and those between generation 0 to generation 3 from Habibi et al. 2024 (C).


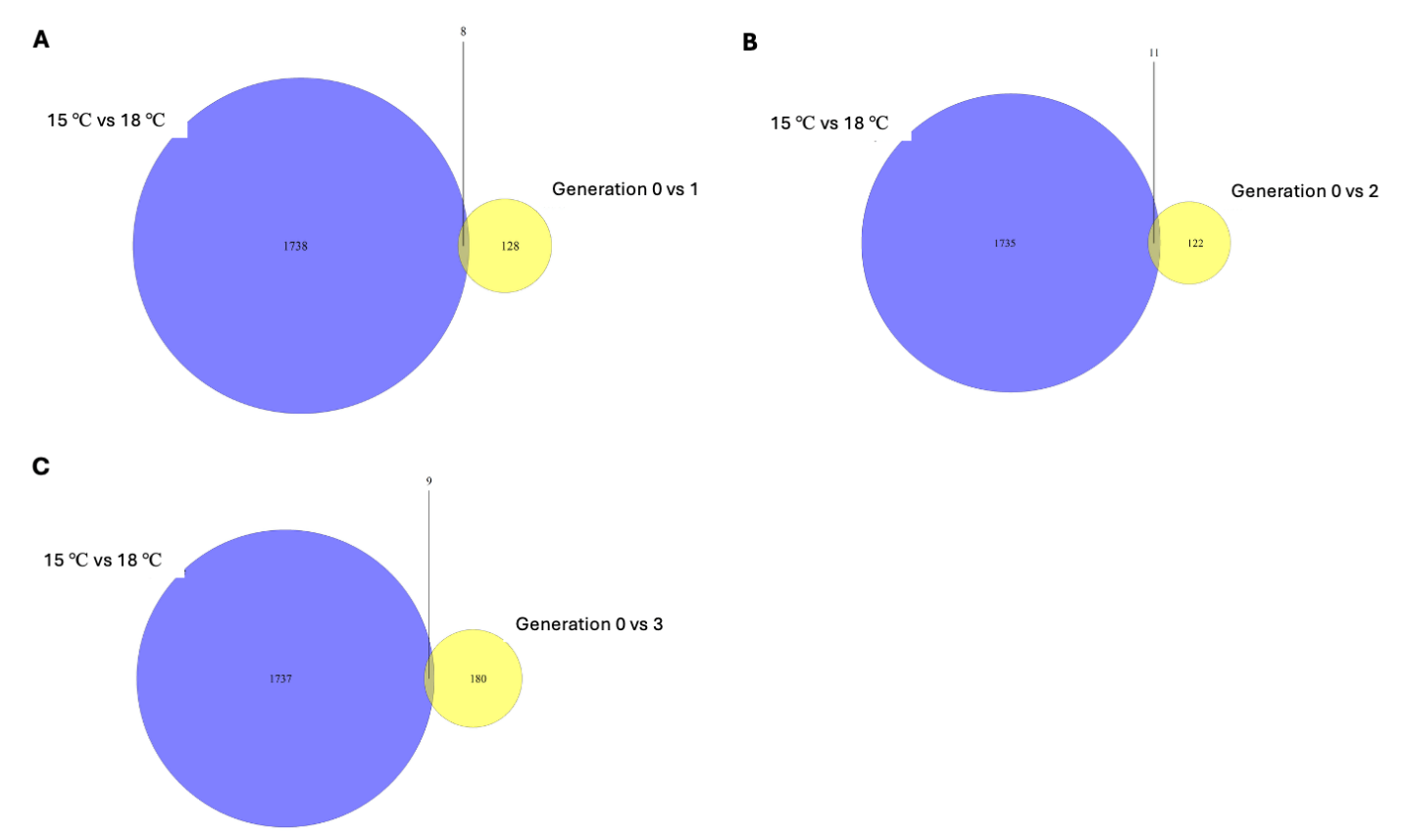


Figure S6: Venn diagram of DMRs due to rearing temperature (blue) found in our study compared to DMRs found in Habibi et al. 2024 (yellow). Overlap of DMRs between fish reared at 15 ℃ vs 18 ℃ and those between generation 0 to generation 1 from Habibi et al. 2024 (A). Overlap of DMRs between fish reared at 15 ℃ vs 18 ℃ and those between generation 0 to generation 2 from Habibi et al. 2024 (B). Overlap of DMRs between fish reared at 15 ℃ vs 18 ℃ and those between generation 0 to generation 3 from Habibi et al. 2024 (C).

Figure S7: Histogram of Weir pairwise F_ST_ (window size of 10,000 bp and a sliding window of 5000 bp) between low and high DI progenitors.

Table S1: HOMER annotation of hypermethylated DMRs due to acclimation temperature. DMRs are annotated based on overlap to TTS (transcription termination site), exons, introns, intergenic, or promoters. The relative enrichment of DMRs in each set of genomic annotations is displayed along with log pvalue (i.e. enrichment of the peaks in promoter regions, exons, etc.)

| **Annotation** | **Number of DMRs** | **Log2 Ratio (obs/exp)** | **LogP enrichment (+values depleted)** |
| --- | --- | --- | --- |
| TTS | 38 | 0.015 | -0.7 |
| Exon | 64 | -0.544 | 7.778 |
| Intron | 399 | 0.279 | -15.982 |
| Intergenic | 181 | -0.281 | 7.11 |
| Promoter | 47 | -0.073 | 0.93 |

Table S2: HOMER annotation of hypomethylated DMRs due to acclimation temperature. DMRs are annotated based on overlap to TTS (transcription termination site), exons, introns, intergenic, or promoters. The relative enrichment of DMRs in each set of genomic annotations is displayed along with log pvalue (i.e. enrichment of the peaks in promoter regions, exons, etc.)

| **Annotation** | **Number of DMRs** | **Log2 Ratio (obs/exp)** | **LogP enrichment (+values depleted)** |
| --- | --- | --- | --- |
| TTS | 61 | 0.218 | -2.056 |
| Exon | 91 | -0.516 | 9.503 |
| Intron | 575 | 0.326 | -29.405 |
| Intergenic | 222 | -0.467 | 20.317 |
| Promoter | 68 | -0.021 | 0.727 |

Table S3: HOMER annotation of hypermethylated DMRs due to domestication index (DI). DMRs are annotated based on overlap to TTS (transcription termination site), exons, introns, intergenic, or promoters. The relative enrichment of DMRs in each set of genomic annotations is displayed along with log pvalue (i.e. enrichment of the peaks in promoter regions, exons, etc.)

| **Annotation** | **Number of DMRs** | **Log2 Ratio (obs/exp)** | **LogP enrichment (+values depleted)** |
| --- | --- | --- | --- |
| TTS | 58 | 0.548 | -5.837 |
| Exon | 61 | -0.69 | 11.342 |
| Intron | 218 | -0.67 | 48.302 |
| Intergenic | 347 | 0.581 | -40.834 |
| Promoter | 85 | 0.705 | -11.673 |

Table S4: HOMER annotation of hypomethylated DMRs due to domestication index (DI). DMRs are annotated based on overlap to TTS (transcription termination site), exons, introns, intergenic, or promoters. The relative enrichment of DMRs in each set of genomic annotations is displayed along with log pvalue (i.e. enrichment of the peaks in promoter regions, exons, etc.)

| **Annotation** | **Number of DMRs** | **Log2 Ratio (obs/exp)** | **LogP enrichment (+values depleted)** |
| --- | --- | --- | --- |
| TTS | 98 | 0.542 | -8.634 |
| Exon | 110 | -0.602 | 14.743 |
| Intron | 415 | -0.504 | 50.965 |
| Intergenic | 606 | 0.622 | -79.143 |
| Promoter | 76 | -0.22 | 2.399 |

Table S5: Genes that showed a significant correlation (with Bonferroni correction applied) between expression levels and percent methylation for DEG and DMRs due to acclimation temperature. Transcript ID, Gene ID, Gene product, and GO ID are described. We also note where the DMR region was located (intron, exon, or promoter region for each gene), whether the correlation was negative or positive. Finally we note the pvalue and adjusted r-2 results of the linear model test for each gene.

| **Transcript ID** | **Gene ID** | **Gene product** | **GO ID/function** | **DMR location** | **Correlation** | **P-value** | **Adjusted r-2** |
| --- | --- | --- | --- | --- | --- | --- | --- |
| XM_047020039.1 | apln | apelin | No GO ID | intron | neg | 2.823004e-05 | 0.381 |
| XM_047020579.1 | mogat2 | monoacylglycerol O-acyltransferase 2 | No GO ID | exon | pos | 3.036420e-06 | 0.4528 |
| XM_047020975.1 | hpda | 4-hydroxyphenylpyruvate dioxygenase a | No GO ID | intron | neg | 2.833100e-06 | 0.4549 |
| XM_047022316.1 | LOC124469183 | LRP2 binding protein | No GO ID | promoter | pos | 3.880637e-09 | 0.6231 |
| XM_047023175.1 | lrrc17 | leucine rich repeat containing 17 | No GO ID | intron | neg | 3.634301e-07 | 0.5138 |
| XM_047022638.1 | cog5 | component of oligomeric golgi complex 5 | No GO ID | intron | neg | 3.549403e-04 | 0.2892 |
| XM_047027751.1 | LOC124472742 | uncharacterized | No GO ID | exon | neg | 4.305805e-06 | 0.4421 |
| XM_047039291.1 | LOC124480190 | WAP four-disulfide core domain protein 2-like | No GO ID | promoter | neg | 2.384565e-04 | 0.3044 |
| XM_047040555.1 | LOC124480897 | ATPase family AAA domain-containing protein 3-like | No GO ID | exon | neg | 6.063330e-05 | 0.3545 |
| XM_047044466.1 | serping1 | serpin peptidase inhibitor, clade G (C1 inhibitor), member 1 | No GO ID | exon | neg | 1.347628e-04 | 0.3256 |
| XM_047044619.1 | rhof | ras homolog family member F | No GO ID | intron | neg | 1.353223e-04 | 0.3255 |
| XR_006957121.1 | LOC124477337 | uncharacterized | No GO ID | exon | pos | 6.602525e-05 | 0.3515 |

Table S6: Genes that showed a significant correlation (with Bonferroni correction applied) between expression levels and percent methylation for DEG and DMRs due to domestication index (DI). Transcript ID, Gene ID, Gene product, and GO ID are described. We also note where the DMR region was located (intron, exon, or promoter region for each gene), whether the correlation was negative or positive. Finally we note the pvalue and adjusted r-2 results of the linear model test for each gene.

| **Transcript ID** | **Gene ID** | **Gene product** | **GO ID/function** | **DMR location** | **Correlation** | **P-value** | **Adjusted r-2** |
| --- | --- | --- | --- | --- | --- | --- | --- |
| XM_047020528.1 | arr3a | arrestin 3a, retinal (X-arrestin) | No GO ID | intron | neg | 1.312e-06 | 0.4778 |
| XM_047025846.1 | LOC124471360 | zinc finger and SCAN domain-containing protein 2-like | No GO ID | exon | neg | 3.854e-05 | 0.3703 |
| XM_047028340.1 | LOC124473082 | beta-1,3-galactosyltransferase 2-like | No GO ID | intron | neg | 0.0002076 | 0.3096 |
| XM_047032478.1 | LOC124475690 | type-2 ice-structuring protein-like | No GO ID | promoter | neg | 0.0001354 | 0.3255 |
| XM_047037577.1 | LOC124479076 | galectin-3-binding protein A-like | No GO ID | intron | neg | 7.447e-08 | 0.5551 |
| XM_047038435.1 | igf2bp1 | insulin-like growth factor 2 mRNA binding protein 1 | No GO ID | intron | neg | 6.419e-05 | 0.3525 |
| XM_047038932.1 | si:dkey-283b1.6 | uncharacterized | No GO ID | intron | neg | 4.635e-10 | 0.6657 |
| XM_047046573.1 | abca12 | ATP-binding cassette, sub-family A (ABC1), member 12 | No GO ID | exon | neg | 0.000298 | 0.2959 |
| XM_047046575.1 | abca12 | ATP-binding cassette, sub-family A (ABC1), member 12 | No GO ID | intron | neg | 1.679e-05 | 0.3985 |
| XM_047049358.1 | gba3 | glucosidase, beta, acid 3, transcript variant X1 | No GO ID | exon | neg | 5.467e-07 | 0.5026 |
| XR_006956573.1 | LOC124471944 | uncharacterized | No GO ID | promoter | neg | 2.825e-07 | 0.5206 |
